# Attachment site selection of life stages of *Ixodes ricinus* ticks on a main large host in Europe, the red deer (*Cervus elaphus*)

**DOI:** 10.1186/s13071-014-0510-x

**Published:** 2014-11-13

**Authors:** Atle Mysterud, Idar Lauge Hatlegjerde, Ole Jakob Sørensen

**Affiliations:** Centre for Ecological and Evolutionary Synthesis (CEES), Department of Biosciences, University of Oslo, P.O. Box 1066 Blindern, NO-0316 Oslo, Norway; Nord-Trøndelag University College, Faculty of Nature Resource Sciences, P.O. Box 2501, NO-7729 Steinkjer, Norway

**Keywords:** *Ixodes ricinus*, Ticks, Tick-borne diseases, Host preferences, Life stages

## Abstract

**Background:**

Ticks and tick-borne diseases are increasing in many areas of Europe and North America due to climate change, while land use and the increased abundances of large hosts play a more controversial role. The pattern of host selection involves a crucial component for tick abundance. While the larvae and nymphs feed on a wide range of different sized hosts, the adult female ticks require blood meal from a large host (>1 kg), typically a deer, to fulfil the life cycle. Understanding the role of different hosts for abundances of ticks is therefore important, and also the extent to which different life stages attach to large hosts.

**Findings:**

We studied attachment site selection of life stages of *I. ricinus* ticks on a main large host in Europe, the red deer (*Cervus elaphus*). We collected from 33 felled red deer pieces of skin from five body parts: leg, groin, neck, back and ear. We counted the number of larval, nymphal, adult male and adult female ticks. Nymphs (42.2%) and adult (48.7%) ticks dominated over larvae (9.1%). There were more larvae on the legs (40.9%), more nymphs on the ears (83.7%), while adults dominated in the groins (89.2%) and neck (94.9%).

**Conclusions:**

Large mammalian hosts are thus a diverse habitat suitable for different life stages of ticks. The attachment site selection reflected the life stages differing ability to move. The spatial separation of life stages may partly limit the role of deer in co-feeding transmission cycles.

## Background

Ticks are known vectors of several pathogens such as *Borrelia burgdorferi* sensu lato causing Lyme disease, the virus (TBEV) causing tick-borne encephalitis, and *Anaplasma phagocytophilum* causing tick-borne fever in livestock, to name some of the more common [1]. The distribution of ticks and its associated diseases is increasing in many areas [2,3], and understanding the mechanisms provide potential for mitigation measures. Some of these increases can be linked to warmer climate, at least at higher elevation and latitudes [4]. However, the topic of how large mammalian hosts and land use affect ticks is heavily debated [5-7]. A crucial component for tick abundance involves finding a suitable host. The larvae and nymphs can feed on a wide range of different sized hosts, but the adult female tick requires a blood meal from a large host (>1 kg) to fulfil the life cycle. Such a large host is, in many systems, typically a deer. The extent to which the dependency for a large host is limiting tick populations is nevertheless unclear, as rodents and birds are likely the more important hosts for larvae [1]. In some ecosystems in the USA, temporal variation in white-tailed deer abundance seemed less important than rodents in driving the abundance of nymphs [8], while in Scotland the abundance of red deer (*Cervus elaphus*) was linked to tick abundances [9]. In Europe, the distribution and density of roe deer (*Capreolus capreolus*) and red deer have markedly increased the last decades [10]. Red deer are considered the most important large host to ticks in many areas of Europe [9,11]. Nevertheless, we are lacking quantitative information on the extent of which red deer may serve different tick life stages. The attachment site selection of different stages on hosts also has importance for co-feeding transmission of diseases such as TBE and several other pathogens [12]. We here provide evidence that different body parts of red deer form different habitats for different life stages of ticks with potential consequences for understanding co-feeding transmission cycles.

## Methods

### Study area

The study area is in Kjølsdalen deer management unit (57.0 km^2^), Eid municipality, Sogn og Fjordane county in south-west of Norway (61°54′ N, 5°59′ E). This is a coastal habitat stretching from sea level up to 500 m above sea level. The habitat is dominated by deciduous forest, mainly alder (*Alnus incana*) at low elevations mixed with aspen (*Populus tremula*) and hazel (*Corylys avellana*), while birch (*Betula* spp.) takes over at higher elevation. There are several plantations of Norway spruce (*Picea abies*) scattered in the area. Agricultural pastures are situated at valley bottoms. The area is densely population by red deer, with no other deer species in the area.

### Red deer data

During the annual autumn harvest in 2013, we collected from 33 felled red deer, pieces of skin from five body parts: leg (below carpus), groin, neck, back and ear. We counted the number of larval, nymphal, adult male and adult female ticks. Most adult males were attached to adult females rather than the skin. The hair was if needed shaved off skin to enhance detection of ticks. We measured size of skin piece to enable calculation of density. We further retrieved date of harvest (Sept. 1^st^ – Oct. 30^th^), elevation at location of harvest (50–500 m a.s.l.), sex and age class (0.5, 1.5 and ≥2.5 years) of the deer. Data were retrieved from 4 male and 4 female calves, 3 male and 7 female yearlings and 7 male and 8 female adults.

### Statistical analyses

We analysed separately the (i) abundance of ticks, (ii) density of ticks and (iii) proportion of life stages of ticks on the different body parts. (i) We analysed number of ticks using a negative binomial error known to fit such data better than Poisson [11,13,14] with individual “ID” as random term using library “glmmADMB” in R vs. 3.1.0. (ii) For analysis of density, we entered size of skin piece as a covariate. (iii) For proportion of life stages, we used first a MANOVA (proportion of all stages as response as a function of body part) and then an ordinary GLMM for each stage. In the GLMM, the response variable was arcsin-sqrt-transformed proportions of larvae, nymphs and adults of total tick count for a given body part with body part as a categorical factor and individual “ID” as a random term using package “lme4”. We weighted the regression with the (sqrt) number of ticks on the body part. For all models, we added sex, age, Julian date of harvest and elevation to the models, but considered sample sizes too small to include interactions. We used a triangle plot to visualize how different proportion of ticks attached to different body parts using library “ade4”.

## Findings

**Figure 1:**
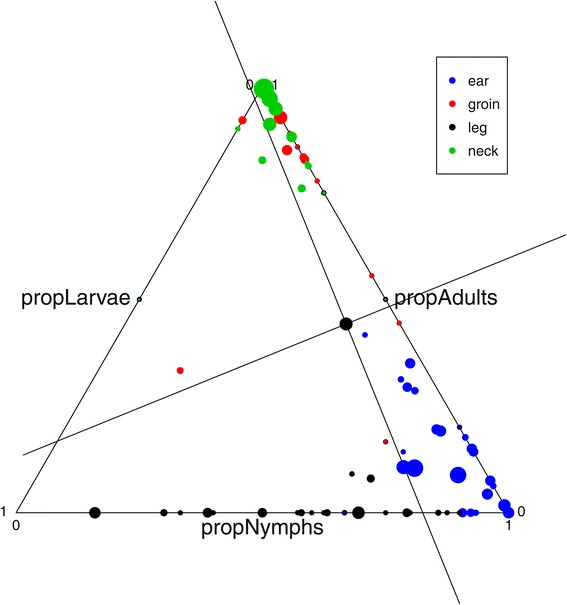
**The proportion of different tick life stages on different body parts of red deer.** Data from the back region was removed due to few ticks. Sizes of points are proportional to (sqrt) total number of ticks.

There were marked differences in abundance, density and proportion of tick life stages depending on the body part (Figure 1, Tables 1 and 2, MANOVA, df = 4, Pillai = 1.308, P = 2.2e^−16^). Adult male and female ticks were mainly found on the neck (94.9%), back (95.0%) and in the groin (89.2%, Table 3A). Nymphs were mainly found on the ears (83.7%) and to some extent also on legs (58.4%, Table 3B). Larvae were mainly found on legs (40.9%, Table 3C). Number of ticks was highest on ears, similar on groin, neck and leg, and lower on the back (Tables 1 and 2A). The higher abundance of ticks on adults compared to calves (Table 2A) was not significant when correcting for size of skin sample, i.e. densities were similar (Table 2B). Density of ticks was higher on the ear (17.3 ticks/dm^2^) than on the leg (7.13 ticks/dm^2^), the groin (5.78 ticks/dm^2^) and the neck (4.97 ticks/dm^2^), with significantly lower density on the back (0.29 ticks/dm^2^, Table 2B). There were fewer ticks and lower density of ticks on red deer harvested late in the fall (Julian date; Sept. 1^st^ – Oct. 30^th^), while elevation did not affect number or density of ticks notably. Effects of sex or age class did not have a significant impact on density (Table 2B).

**Table 1:** **An overview of tick life stage placement on different body parts of red deer (n = 33), Norway**

	**Ear**	**Neck**	**Leg**	**Groin**	**Back**	**Total**
Larvae	54	7	180	13	2	256
Nymphs	848	31	257	50	0	1186
Adult males	38	326	1	200	14	579
Adult females	73	374	2	319	24	792
Larvae (%)	5.3	0.9	40.9	2.2	5.0	9.1
Nymphs (%)	83.7	4.2	58.4	8.6	0	42.2
Adults (%)	11.0	94.9	0.7	89.2	95.0	48.7
Size of skin piece (dm^2^)	1.77	4.50	1.87	3.05	4.14	
Density of all ticks (/dm^2^)	17.3	4.97	7.13	5.78	0.29

**Table 2:** **Parameter estimates and test statistics from mixed effects models with negative binomial error for the relationship between (A) abundance and (B) density of ticks on red deer, Norway**

**Parameter**	**Estimate**	**Std. Error**	**z value**	**P**
**A. Abundance**				
Intercept	7.3617	2.1670	3.400	0.001
Bodypart (groin vs leg)	0.3586	0.2198	1.630	0.103
Bodypart (neck vs. leg)	0.1177	0.2221	0.530	0.596
Bodypart (ear vs. leg)	1.0613	0.2200	4.820	<0.001
Bodypart (back vs. leg)	−2.7549	0.2931	−9.400	<0.001
Sex (f vs. m)	−0.4887	0.2862	−1.710	0.088
Age (1.5 vs. 0.5)	0.6453	0.3802	1.700	0.090
Age (≥2.5 vs. 0.5	0.7498	0.3507	2.140	0.033
Julian date	−0.0189	0.0079	−2.380	0.017
Elevation	−0.0015	0.0011	−1.370	0.170
**B. Density**				
Intercept	5.3200	1.9700	2.710	0.007
Size of skin	2.24e-05	4.26e-06	5.260	<0.001
Bodypart (groin vs leg)	0.0435	0.2030	0.210	0.830
Bodypart (neck vs. leg)	−0.6670	0.2410	−2.760	0.006
Bodypart (ear vs. leg)	1.0500	0.1950	5.370	<0.001
Bodypart (back vs. leg)	−3.1800	0.2890	−11.010	<0.001
Sex (f vs. m)	−0.2120	0.2590	−0.820	0.414
Age (1.5 vs. 0.5)	0.4900	0.3390	1.440	0.149
Age (≥2.5 vs. 0.5	0.5640	0.3140	1.800	0.072
Julian date	−0.0136	0.0071	−1.910	0.056
Elevation	−0.0006	0.0010	−0.620	0.533

**Table 3:** **Parameter estimates from mixed effects models with (arcsin[sqrt]) proportion of (A) larvae, (B) nymphs, and (C) adult ticks as response ticks on red deer, Norway**

**Parameter**	**Estimate**	**SE**	**Lower 95% CL**	**Upper 95% CL**
**A. Larvae**				
Intercept	0.6109	0.0402	0.5305	0.6913
Bodypart (groin vs. leg)	−0.5594	0.0527	−0.6647	−0.4541
Bodypart (neck vs. leg)	−0.5670	0.0541	−0.6752	−0.4588
Bodypart (ear vs. leg)	−0.4536	0.0496	−0.5527	−0.3544
Bodypart (back vs. leg)	−0.4508	0.1075	−0.6658	−0.2358
**B. Nymphs**				
Intercept	0.9479	0.0478	0.8524	1.0434
Bodypart (groin vs. leg)	−0.7018	0.0646	−0.8310	−0.5727
Bodypart (neck vs. leg)	−0.7835	0.0661	−0.9157	−0.6512
Bodypart (ear vs. leg)	0.2680	0.0607	0.1466	0.3894
Bodypart (back vs. leg)	−0.9450	0.1310	−1.2071	−0.6830
**C. Adults**				
Intercept	0.0230	0.0447	−0.0664	0.1124
Bodypart (groin vs. leg)	1.2677	0.0608	1.1461	1.3894
Bodypart (neck vs. leg)	1.3564	0.0622	1.2319	1.4809
Bodypart (ear vs. leg)	0.2526	0.0572	0.1382	0.3669
Bodypart (back vs. leg)	1.3816	0.1232	1.1352	1.6280

Individual ID of red deer was a random term in the models.

## Discussion

The role of large sized hosts for determining tick abundances and their role in the transmission cycles of disease remain controversial [7,15,16]. We provide insight on how different stages of ticks select different body parts for attachment on a main large host in Europe, the red deer. The attachment site selection reflected the life stages differing ability to move. The larvae were mainly found on legs and ears with a short distance to move after encountering the host, with nymphs mainly on ears and some on legs, while adult ticks moved longer distances to the neck and groin region. Deer species are most often discussed in general terms when it comes to their impact on tick ecology. However, deer species may differ in importance as hosts for ticks for a number of reasons, and patterns of attachment site selection of ticks may also differ among deer species. Larger species or age classes of deer would mean longer distances to move to reach a given location for a tick entering the leg or head. A study on attachment site selection on roe deer reported similarly that larvae mainly attached to legs (and head), nymphs on the head and adults on neck [12]. However, fewer adult ticks were found on ears of roe deer and red deer calves compared to adults [17]. Further, feeding niches of deer species differ. We would expect large browsers such as moose (*Alces alces*) to have fewer ticks on the head due to more feeding on trees and less on ground vegetation and thus a lower opportunity for ticks to enter the head. Indeed, fewer ticks and a higher proportion of adult ticks and lower proportion of larval and nymphal ticks were found on moose ears compared to ears from red deer and roe deer [17]. Deer ears are often used in tick burden studies due to ease of sampling by hunters [17], but our study also highlights that sampling of ears may give biased estimates of the overall proportion of tick life stages. Our data derive from sampling in Autumn. Similar studies in the main questing period in early summer may affect proportions of life stages if questing times differ [18], but are unlikely to affect attachment site selection. Co-feeding of larval and nymphal ticks is important for transmission of TBE and may occur for several other pathogens [19]. The partial spatial separation of tick stages on deer may partly limit the opportunity for such transmission [12], though legs and ears do have both larvae and nymphs. Our study thus has potential implications for understanding tick disease transmission, and highlights and suggests that more studies are needed for understanding how different species of deer function as hosts for ticks.
